# Supplementary material for: Characterization of Bioactive Compounds in Spent Mushroom Substrate: A Metabolomic Perspective on Its Untapped Potential
Source: Foods. 2025 Dec 30;15(1):109. doi: 10.3390/foods15010109 (PMC12785439; doi:10.3390/foods15010109)
Supplement: Supplementary file 1 [file foods-15-00109-s001.zip › foods-supplemental-20250523.pdf]

Table S1. Content of ergothioneine sulfonate in spent pink oyster mushroom substrate, spent pearl oyster mushroom substrate, and blank substrate.

	**Content of ergothioneine sulfonate (μg·g^{-1})**				
	**Pink Oyster Mushroom Substrate**		**Pearl Oyster Mushroom Substrate**		**Blank substrate**
	Surface of substrate	Core of substrate	Surface of substrate	Core of substrate	
First harvest	13.94±6.39 ^{A}	24.82±11.06 ^{A}	11.03±5.22 ^{A}	0.11±0.08 ^{A}	
Second harvest	16.02±8.16 ^{A,B}	27.65±4.41 ^{A}	5.79±1.65 ^{B}	0.46±0.18 ^{B}	<0.007 nM
Third harvest	10.04±7.81 ^{A}	11.10±4.04 ^{A}	-	-	

Values are mean ± S.E.M. (n=5). The results are not significantly different down the column, while within each row, values with different uppercase superscript letters (A, B, C) are significantly different (P<0.05, ANOVA, Bonferroni Test).

Table S2. Content of ergothioneine sulfonate in pink oyster mushroom and pearl oyster mushroom.

	**Content of ETSO$_3$H (μg·g^{-1})**	
	**Pink Oyster Mushroom**	**Pearl Oyster Mushroom**
First harvest	0.23±0.03 ^{a,A}	11.60±2.95 ^{a,B}
Second harvest	0.25±0.02 ^{a,A}	5.47±1.83 ^{a,B}
Third harvest	0.61±0.10 ^{b}	-

Values are mean ± S.E.M. (n=5). Within each column, values with different lowercase superscript letters (a, b, c) are significantly different (P<0.05, ANOVA, Bonferroni Test), while within each row, values with different uppercase superscript letters (A, B, C) are significantly different (P<0.05, ANOVA, Bonferroni Test).

Table S3. Content of Carbon, hydrogen, nitrogen, and protein in spent pink oyster mushroom substrate, spent pearl oyster mushroom substrate, and blank substrate.

	**Pink Oyster Mushroom Substrate**						**Pearl Oyster Mushroom Substrate**				**Blank substrate**
	Surface of substrate			Core of substrate			Surface of substrate		Core of substrate		
	first harvest	second harvest	third harvest	first harvest	second harvest	third harvest	first harvest	second harvest	first harvest	second harvest	
C	44.96±0.40	45.01±0.42	44.69±0.25	46.02±0.20	46.09±0.35	45.48±0.12	44.51±0.48	44.68±0.45	45.65±0.17	45.22±0.24	45.75±0.23
H	5.79±0.10	5.73±0.06	5.87±0.12	5.96±0.10	5.87±0.08	5.83±0.06	5.88±0.05	5.89±0.04	5.78±0.06	5.83±0.02	6.00±0.03
N	0.87±0.06	0.70±0.03	0.80±0.07	0.79±0.08	0.57±0.06	0.68±0.04	0.76±0.07	0.77±0.07	0.69±0.04	0.80±0.05	0.68±0.09
protein	5.41±0.41	4.38±0.18	4.99±0.45	4.96±0.51	3.55±0.35	4.28±0.24	4.74±0.45	4.79±0.41	4.30±0.25	4.95±0.32	4.24±0.57

Values are mean ± S.E.M. (n=5). The results are not significantly different across the rows.

Table S4 . Content of Carbon, hydrogen, and nitrogen in pink oyster mushroom fruiting, pearl oyster mushroom fruiting.

	**Pink Oyster Mushroom**			**Pearl Oyster Mushroom**	
	first harvest	second harvest	third harvest	first harvest	second harvest
C	39.53±0.36 ^{a}	39.95±0.12 ^{a}	39.44±0.24 ^{a}	39.49±0.10 ^{a}	40.16±0.25 ^{a}
H	6.29±0.04 ^{a}	6.51±0.10 ^{a}	6.50±0.09 ^{a}	6.30±0.05 ^{a}	6.61±0.10 ^{a}
N	4.48±0.38 ^{a}	4.07±0.16 ^{a}	3.55±0.26 ^{a}	4.62±0.11 ^{a}	4.52±0.36 ^{a}
S	0.13±0.01 ^{a,b}	0.12±0.01 ^{a,b}	0.11±0.01 ^{b}	0.13±0.01 ^{a,b}	0.17±0.02 ^{a}
protein	28.02±2.36 ^{a}	25.44±1.01 ^{a}	22.19±1.65 ^{a}	28.88±0.71 ^{a}	28.25±2.24 ^{a}

Values are mean ± S.E.M. (n=5). Means across the column with different letters (a, b) were significantly different ($P<0.05$, ANOVA, Bonferroni Test).

Table S5. List of 20 compounds identified in different types of SMS with significant differences from the blank substrate, excluding those detected in all four groups.

Accepted Description	m/z	Retention time (min)	Adducts	Formula	Mass Error (ppm)	Fold Change									
						Spent Pinkl Oyster Mushroom Substrate						Spent Pearl Oyster Mushroom Substrate			
						Surface of substrate			Core of substrate			Surface of substrate		Core of substrate	
						First harvest	Second harvest	Third harvest	First harvest	Second harvest	Third harvest	First harvest	Second harvest	First harvest	Second harvest
**Compounds only different in Surface of Spent Pearl Oyster Mushroom Substrate**															
o-Hydroxycinnamaldehyde	166.0874	1.06	M+H-H_2O, M+H, M+NH_4	$C_9H_8O_2$	7.88	-	-	-	-	-	-	75.71	102.08	-	-
Glycerophosphocholine	258.1098	0.49	M+H	$C_8H_{20}NO_6P$	-1.35	-	-	-	-	-	-	-2.84	3.20	-	-
Sepiapterin	238.0938	0.51	M+H-H_2O, M+H, M+K	$C_9H_{11}N_5O_3$	1.51	-	-	-	-	-	-	33.89	3.20	-	-
Maltotriose	527.1583	0.51	M+Na	$C_{18}H_{32}O_{16}$	0.18	-	-	-	-	-	-	-2.50	5.22	-	-
Stachyose	689.2096	0.50	M+Na	$C_{18}H_{32}O_{16}$	0.18	-	-	-	-	-	-	-2.83	4.84	-	-
(4R,5S,6S,7S,9R,11E,13E,16R)-7-Ethyl-4,6-dihydroxy-5-methoxy-9,16-dimethyloxa	351.214	4.03	M+H-H_2O, M+K	$C_{20}H_{32}O_6$	-7.02	-	-	-	-	-	-	1.79	2.30	-	-

cyclohexadeca-11,13-diene-2,10-dione															
**Compounds only different in Core of Spent Pearl Oyster Mushroom Substrate**															
Hovenine A	487.3325	3.96	M+H	$C_{27}H_{42}N_4O_4$	9.54	-	-	-	-	-	-	-	-	55.31	48.29
Citbismine E	653.2155	3.48	M+H-H_2O	$C_{36}H_{34}N_2O_{11}$	3.80	-	-	-	-	-	-	-	-	-49.69	-21.95
Vargulin	423.2655	4.19	M+NH_4	$C_{22}H_{27}N_7O$	9.82	-	-	-	-	-	-	-	-	2.32	2.22
**Compounds only different in Core of Spent Pink Oyster Mushroom Substrate**															
6-Hydroxymellein	195.0666	3.02	M+H, M+Na	$C_{10}H_{10}O_4$	7.38	-	-	-	53.18	87.70	67.20	-	-	-	-
2'-Hydroxy-5'-methylacetophenone	151.0763	2.65	M+H	$C_9H_{10}O_2$	6.41	-	-	-	176.33	312.47	338.45	-	-	-	-
(-)-Farnesiferol C	365.2143	3.42	M+H-H_2O, M+H, M+Na	$C_{24}H_{30}O_4$	8.37	-	-	-	5.25	6.87	6.27	-	-	-	-
Tetrahydropteroyltri-L-glutamate	609.227	3.61	M+H-H_2O	$C_{24}H_{34}N_8O_{12}$	1.07	-	-	-	-42.06	-51.51	-62.41	-	-	-	-
**Compounds both different in Core of Spent Pink Oyster Mushroom Substrate and Spent Pearl Oyster Mushroom Substrate**															

(±)-Naringenin	273.0769	3.14	M+H	$C_{15}H_{12}O_5$	4.07	-	-	-	-449.47	-1157.24	-416.18	-	-	-1036.26	-542.35
**Compounds both different in Surface and Core of Spent Pearl Oyster Mushroom Substrate**															
Oleamide	282.2792	5.44	M+H	$C_{18}H_{35}NO$	0.36	-	-	-	-	-	-	-1.96	-2.29	-1.84	-3.12
12-oxo,13-hydroxy-9Z-octadecenoic acid	335.2188	4.39	M+Na	$C_{18}H_{32}O_4$	-1.52	-	-	-	-	-	-	3.69	3.82	3.26	1.49
3-PT-PtdIns (3,4,5)-P3 (1,2-dioctanoyl)	843.1593	2.30	M+H, M+Na	$C_{25}H_{50}O_{21}P_4S$	0.54	-	-	-	-	-	-	-6776.94	-462.08	-5847.15	-403.52
**Compounds different in Surface of Spent Pearl Oyster Mushroom Substrate, and Surface and Core of Spent Pink Mushroom Substrate**															
1-Linoleoylglycerophosphocholine	520.338	4.53	M+H	$C_{26}H_{50}NO_7P$	-3.41	-4.98	-9.55	-4.89	-2.65	-2.50	-4.23	-6.02	-4.98	-	-
**Compounds different in Core of Spent Pearl Oyster Mushroom Substrate, and Surface and Core of Spent Pink Mushroom Substrate**															
Mangiferin	423.0934	2.42	M+H-H_2O, M+H,	$C_{19}H_{18}O_{11}$	2.94	-179.97	-103.76	-82.73	-84.35	-55.19	-97.21	-	-	-88.16	-74.82

			M+Na, M+K												
4R-aminopentanoic acid	118.0868	0.50	M+H	$C_5H_{11}NO_2$	4.60	-35.93	-96.57	-185.08	-399.83	-1370.72	-599.19	-	-	-556.26	-935.61

Table S6. 38 compounds in Spent Pearl Oyster Mushroom Substrate with significant differences compared to the blank substrate.

Accepted Description	m/z	Retention Time (min)	Adducts	Formula	Mass Error (ppm)	Fold Change			
						Surface of substrate		Core of substrate	
						First harvest	Second harvest	First harvest	Second harvest
**Polyphenol**									
Kuwanon V	685.2179	0.50	M+K	$C_{40}H_{38}O_8$	-2.98208	-9.96	-9.93	-6.06	-11.28
Morin	303.0521	2.42	M+H-H2O, M+H	$C_{15}H_{10}O_7$	7.214998	-361.16	-132.14	-76.13	-48.19
6a,7-Dihydroxymaackiain	317.0671	2.54	M+H-H2O, M+H	$C_{16}H_{12}O_7$	4.836982	-533.60	-124.85	-51.02	-30.00
Taxifolin 3-arabinoside	437.1078	2.54	M+H-H2O, M+H, M+Na	$C_{20}H_{20}O_{11}$	0.007846	-141.61	-51.84	-44.91	-33.89
Trifolirhizin	447.1264	2.87	M+H-H2O, M+H, M+Na	$C_{22}H_{22}O_{10}$	-4.81944	-13.19	-8.43	-3.58	-2.88
Isomangiferin	405.0854	2.92	M+H-H2O	$C_{19}H_{18}O_{11}$	8.935896	-35.45	-18.78	-17.48	-12.21

Glycitin	447.1276	2.94	M+H-H2O, M+H, M+Na	$C_{22}H_{22}O_{10}$	-2.2116	-35.68	-22.46	-7.34	-5.80
Dalpanol O-glucoside	597.1922	3.06	M+H-H2O, M+Na, M+K	$C_{29}H_{34}O_{12}$	-3.59762	-68.23	-13.96	-10.76	-5.34
Kuwanone G	715.215	3.07	M+Na	$C_{40}H_{36}O_{11}$	-0.02568	-9.86	-9.83	-5.71	-3.89
Macaflavone II	435.1787	3.27	M+H-H2O, M+H, M+Na	$C_{26}H_{26}O_{6}$	-3.41565	-30.07	-15.40	-11.74	-7.70
Icaritin	387.14	3.65	M+H-H2O, M+H, M+K	$C_{21}H_{22}O_{7}$	-9.9026	-18.69	-13.41	-12.61	-6.99
Pelargonidin	271.0619	3.49	M+H	$C_{15}H_{10}O_{5}$	6.492701	-584.09	-1267.52	-584.65	-326.97
5-(Hydroxyphenyl)-γ-valerolactone-O-sulphate	273.0417	2.42	M+H-H2O, M+H	$C_{11}H_{12}O_{6}S$	-3.82018	-3424.34	-343.17	-235.46	-116.90
(4E)-5-(4-Methoxyphenyl)-2-methyl-3-oxo-4-penten-1-yl hydrogen sulfate	301.0741	3.20	M+H, M+Na	$C_{13}H_{16}O_{6}S$	0.110528	-22.41	-32.23	-31.28	-28.89
Alantolactone	233.1549	4.62	M+H, M+Na	$C_{15}H_{20}O_{2}$	5.598498	-38.08	-50.90	-53.21	-49.50
**Lipids and Lipid Derivatives**									
3-[(3-Hydroxytridecanoyl)oxy]-4-(trimethylammonio)butanoate	396.2689	3.83	M+Na	$C_{20}H_{39}NO_{5}$	-8.32589	9.59	9.20	11.49	10.59
Oleamide	282.2792	5.44	M+H	$C_{18}H_{35}NO$	0.365465	-1.96	-2.29	-1.84	-3.12
PC(16:0/0:0)[U] / PC(16:0/0:0)[rac]	496.3381	4.62	M+H	$C_{24}H_{50}NO_{7}P$	-3.273	-7.59	-11.34	-1.95	-2.13
PI(22:0/20:5(5Z,8Z,11Z,14Z,17Z))	963.5956	8.30	M+NH4, M+Na, M+K	$C_{51}H_{89}O_{13}P$	2.461271	-189.61	-683.11	-69.73	-76.78
PI(22:0/18:3(6Z,9Z,12Z))	939.5959	8.95	M+NH4, M+Na, M+K	$C_{49}H_{89}O_{13}P$	2.829961	-8.46	-9.13	-4.02	-5.87
PI(22:0/20:4(5Z,8Z,11Z,14Z))	965.6096	9.12	M+NH4, M+Na	$C_{51}H_{91}O_{13}P$	0.662973	-454.77	-761.36	-34.86	-62.54

MGDG(18:2(9Z,12Z)/18:2(9Z,12Z))	801.5521	9.55	M+Na	$C_{45}H_{78}O_{10}$	4.311619	-16.41	-17.63	-14.06	-16.09
**Terpenoids**									
Harrisonin	539.1874	2.66	M+Na, M+K	$C_{27}H_{32}O_{10}$	-2.70702	-296.55	-55.08	-44.81	-28.73
16-Methoxy-2,3-dihydro-3-hydroxytabersonine	385.2121	2.87	M+H	$C_{22}H_{28}N_2O_4$	-0.30091	-60.58	-49.96	-41.03	-37.11
Ganoderenic acid D	513.2873	4.09	M+H	$C_{30}H_{40}O_7$	5.024635	21.62	29.81	22.69	24.31
Minabeolide-8	471.3141	4.08	M+H-H_2O, M+H	$C_{29}H_{42}O_5$	7.595305	9.95	11.58	11.44	11.22
2-[(3S,3aR,5R,8R,8aS)-3,8-Dihydroxy-8-(hydroxymethyl)-3-methyl-2-oxodecahydroazulen-5-yl]propan-2-yl β-D-glucopyranoside	487.1942	3.24	M+K	$C_{21}H_{36}O_{10}$	0.498303	-40.23	-18.80	-21.69	-11.98
Hexandraside D	805.2945	3.52	M+H-H_2O	$C_{39}H_{50}O_{19}$	3.872006	-206.06	-73.52	-89.23	-22.40
**Steroids and Vitamin D Derivatives**									
1α,25-dihydroxy-24a-homo-26,27-dimethyl-22-thiavitamin D3 / 1α,25-dihydroxy-24a-homo-26,27-dimethyl-22-thiacholecalciferol	515.2982	3.97	M+Na, M+K	$C_{29}H_{48}O_3S$	5.601362	8.50	11.25	9.28	9.46
2-deoxy-20-hydroxy-5alpha-ecdysone 3-acetate	489.3212	3.97	M+H-H_2O	$C_{29}H_{46}O_7$	0.246843	42.47	57.03	50.28	50.01
(23R,25R)-1α,25-dihydroxyvitamin D3 26,23-lactone / (23R,25R)-1α,25-dihydroxycholecalciferol 26,23-lactone	427.2833	4.07	M+H-H_2O, M+H	$C_{27}H_{40}O_5$	-2.1415	7.12	8.59	8.13	7.71
Tacalcitol	455.2919	4.10	M+K	$C_{27}H_{44}O_3$	-0.62329	5.36	6.84	5.64	5.96

**Tetrapyrroles and Related Cofactors**									
Sirohydrochlorin	863.3023	3.79	M+H	$C_{42}H_{46}N_4O_{16}$	4.790577	-30.34	-59.92	-85.57	-36.57
Precorrin 1	833.2924	3.84	M+H-H_2O, M+Na	$C_{41}H_{46}N_4O_{16}$	5.604641	-30.34	-54.54	-27.32	-17.67
**Fatty Acid Derivatives**									
12-oxo,13-hydroxy-9Z-octadecenoic acid	335.2188	4.39	M+Na	$C_{18}H_{32}O_4$	-1.521	3.69	3.82	3.26	1.49
Leukotriene D4 methyl ester	511.2812	4.14	M+H-H_2O, M+H	$C_{26}H_{42}N_2O_6S$	-4.81183	8.61	10.65	9.68	10.58
**Other Categories**									
4R-aminopentanoic acid	118.0868	0.50	M+H	$C_5H_{11}NO_2$	4.595853	-135.23	-166.28	-556.26	-935.61
3-PT-PtdIns (3,4,5)-P3 (1,2-dioctanoyl)	843.1593	2.30	M+H, M+Na	$C_{25}H_{50}O_{21}P_4S$	0.538761	-6776.94	-539.10	-5847.15	-403.52
Cerebroside B	750.5449	7.89	M+H, M+Na	$C_{41}H_{77}NO_9$	-5.75781	9.63	21.89	9.14	6.67
N-docosahexaenoyl glutamine	457.3052	4.09	M+H-H_2O, M+H	$C_{27}H_{40}N_2O_4$	-1.85897	10.53	13.32	11.06	11.06
(1S,2S,3R,4R,5R,6S)-2,3,4,5,6-Pentahydroxycyclohexyl α-D-galactopyranosyl-(1->6)-α-D-galactopyranosyl-(1->6)-α-D-galactopyranoside	667.2331	3.72	M+H	$C_{24}H_{42}O_{21}$	5.900476	-107.66	-101.38	-88.84	-41.20
Phosphopantetheine	381.0822	0.48	M+Na, M+K	$C_{11}H_{23}N_2O_7PS$	-9.48272	-3.78	-5.34	-2.25	-4.53

Table S7. 38 compounds in Spent Pink Oyster Mushroom Substrate with significant differences compared to the blank substrate.

Accepted Description	m/z	Retention time (min)	Adducts	Formula	Mass Error (ppm)	Fold Change					
						Surface of substrate			Core of substrate		
						First harvest	Second harvest	Third harvest	First harvest	Second harvest	Third harvest
**Polyphenol**											
Kuwanon V	685.2179	0.50	M+K	$C_{40}H_{38}O_8$	-2.98208	-12.22	-12.85	-11.10	-7.55	-10.11	-11.02
Morin	303.0521	2.42	M+H-H_2O, M+H	$C_{15}H_{10}O_7$	7.214998	-369.03	-77.80	-59.54	-55.09	-39.23	-54.50
6a,7-Dihydroxymaackiain	317.0671	2.54	M+H-H_2O, M+H	$C_{16}H_{12}O_7$	4.836982	-1298.94	-63.75	-49.64	-52.65	-48.81	-60.05
Taxifolin 3-arabinoside	437.1078	2.54	M+H-H_2O, M+H, M+Na	$C_{20}H_{20}O_{11}$	0.007846	-292.62	-50.45	-35.89	-40.34	-31.68	-44.09
Trifolirhizin	447.1264	2.87	M+H-H_2O, M+H, M+Na	$C_{22}H_{22}O_{10}$	-4.81944	-10.55	-6.08	-5.99	-4.08	-2.89	-3.78
Isomangiferin	405.0854	2.92	M+H-H_2O	$C_{19}H_{18}O_{11}$	8.935896	-48.20	-16.22	-11.35	-14.18	-15.06	-18.96
Glycitin	447.1276	2.94	M+H-H_2O, M+H, M+Na	$C_{22}H_{22}O_{10}$	-2.2116	-19.24	-13.71	-17.51	-8.00	-4.90	-6.34
Dalpanol O-glucoside	597.1922	3.06	M+H-H_2O, M+Na, M+K	$C_{29}H_{34}O_{12}$	-3.59762	-276.85	-10.96	-7.90	-10.71	-11.66	-14.68
Kuwanone G	715.215	3.07	M+Na	$C_{40}H_{36}O_{11}$	-0.02568	-27.51	-6.84	-5.98	-6.03	-4.90	-5.18
Macaflavone II	435.1787	3.27	M+H-H_2O,	$C_{26}H_{26}O_6$	-3.41565	-27.18	-10.27	-8.76	-9.42	-8.07	-9.41

			M+H, M+Na								
Icaritin	387.14	3.65	M+H-H2O, M+H, M+K	$C_{21}H_{22}O_7$	-9.9026	-32.66	-13.03	-10.23	-15.03	-10.20	-11.04
Pelargonidin	271.0619	3.49	M+H	$C_{15}H_{10}O_5$	6.492701	-981.43	-1747.54	-4929.58	-765.98	-877.38	-918.96
Mangiferin	423.0934	2.42	M+H-H2O, M+H, M+Na, M+K	$C_{19}H_{18}O_{11}$	2.941711	-179.97	-103.76	-82.73	-84.35	-55.19	-97.21
5-(Hydroxyphenyl)-γ-valerolactone-O-sulphate	273.0417	2.42	M+H-H2O, M+H	$C_{11}H_{12}O_6S$	-3.82018	-1929.26	-200.83	-202.84	-164.68	-95.70	-166.69
(4E)-5-(4-Methoxyphenyl)-2-methyl-3-oxo-4-penten-1-yl hydrogen sulfate	301.0741	3.20	M+H, M+Na	$C_{13}H_{16}O_6S$	0.110528	-31.30	-26.97	-28.75	-25.05	-33.85	-32.05
Alantolactone	233.1549	4.62	M+H, M+Na	$C_{15}H_{20}O_2$	5.598498	-34.29	-30.63	-44.00	-27.40	-24.33	-19.49
4,5-Dimethoxy-1,2-benzenedicarboxylic acid	209.046	2.40	M+H-H2O, M+H, M+Na	$C_{10}H_{10}O_6$	6.952475	41.27	38.36	56.59	64.36	114.66	90.71
**Lipids and Lipid Derivatives**											
3-[(3-Hydroxytridecanoyl)oxy]-4-(trimethylammonio)butanoate	396.2689	3.83	M+Na	$C_{20}H_{39}NO_5$	-8.32589	10.39	11.09	13.13	10.52	14.41	12.76
PC(16:0/0:0)[U] / PC(16:0/0:0)[rac]	496.3381	4.61	M+H	$C_{24}H_{50}NO_7P$	-3.273	-6.97	-14.23	-10.67	-2.95	-2.98	-5.10
MGDG(18:2(9Z,12Z)/18:2(9Z,12Z)	801.5521	9.55	M+Na	$C_{45}H_{78}O_{10}$	4.311619	-15.05	-16.91	-19.92	-16.89	-15.84	-15.60

)											
PI(22:0/20:4(5Z,8Z,11Z,14Z))	965.6096	9.12	M+NH4, M+Na	$C_{51}H_{91}O_{13}P$	0.662973	-96.28	-101.13	-143.14	-46.66	-81.97	-115.61
PI(22:0/18:3(6Z,9Z,12Z))	939.5959	8.95	M+NH4, M+Na, M+K	$C_{49}H_{89}O_{13}P$	2.829961	-13.34	-8.06	-4.77	-12.66	-12.20	-7.54
PI(22:0/20:5(5Z,8Z,11Z,14Z,17Z))	963.5956	8.30	M+NH4, M+Na, M+K	$C_{51}H_{89}O_{13}P$	2.461271	-245.71	-232.23	-270.70	-81.82	-117.42	-152.78
1-Linoleoylglycerophosphocholine	520.338	4.53	M+H	$C_{26}H_{50}NO_7P$	-3.40816	-4.98	-9.55	-4.89	-2.65	-2.50	-4.23
**Terpenoids**											
Harrisonin	539.1874	2.66	M+Na, M+K	$C_{27}H_{32}O_{10}$	-2.70702	-1034.21	-55.00	-35.76	-46.08	-64.83	-76.76
16-Methoxy-2,3-dihydro-3-hydroxytabersonine	385.2121	2.87	M+H	$C_{22}H_{28}N_2O_4$	-0.30091	-47.36	-39.20	-45.60	-41.32	-35.34	-49.05
Ganoderenic acid D	513.2873	4.09	M+H	$C_{30}H_{40}O_7$	5.024635	15.82	26.70	38.90	17.77	24.94	27.22
Minabeolide-8	471.3141	4.08	M+H-H2O, M+H	$C_{29}H_{42}O_5$	7.595305	7.99	11.15	14.44	8.56	11.97	12.08
2-[(3S,3aR,5R,8R,8aS)-3,8-Dihydroxy-8-(hydroxymethyl)-3-methyl-2-oxodecahydroazulen-5-yl]propan-2-yl β-D-glucopyranoside	487.1942	3.24	M+K	$C_{21}H_{36}O_{10}$	0.498303	-23.24	-13.79	-13.11	-12.88	-9.62	-9.56
Hexandraside D	805.2945	3.52	M+H-H_2O	$C_{39}H_{50}O_{19}$	3.872006	-936.60	-36.49	-31.10	-71.52	-73.87	-67.718

**Steroids and Vitamin D Derivatives**											
1α,25-dihydroxy-24a-homo-26,27-dimethyl-22-thiavitamin D3 / 1α,25-dihydroxy-24a-homo-26,27-dimethyl-22-thiacholecalciferol	515.2982	3.97	M+Na, M+K	$C_{29}H_{48}O_3S$	5.601362	7.12	10.31	13.79	7.32	10.52	10.91
2-deoxy-20-hydroxy-5alpha-ecdysone 3-acetate	489.3212	3.97	M+H-H_2O	$C_{29}H_{46}O_7$	0.246843	34.69	52.45	73.18	39.15	59.35	58.05
(23R,25R)-1α,25-dihydroxyvitamin D3 26,23-lactone / (23R,25R)-1α,25-dihydroxycholecalciferol 26,23-lactone	427.2833	4.07	M+H-H_2O, M+H	$C_{27}H_{40}O_5$	-2.1415	7.89	8.98	10.35	6.50	9.30	9.28
Tacalcitol	455.2919	4.10	M+K	$C_{27}H_{44}O_3$	-0.62329	4.19	6.56	9.01	4.69	6.39	6.70
**Tetrapyrroles and Related Cofactors**											
Sirohydrochlorin	863.3023	3.79	M+H	$C_{42}H_{46}N_4O_{16}$	4.790577	-1660.60	-61.10	-46.90	-99.83	-120.18	-91.79
Precorrin 1	833.2924	3.84	M+H-H_2O, M+Na	$C_{41}H_{46}N_4O_{16}$	5.604641	-267.07	-27.94	-22.39	-40.35	-37.35	-39.86
Adenosine	268.1056	0.67	M+H	$C_{10}H_{13}N_5O_4$	6.041918	8.61	4.33	3.34	4.23	3.59	2.96
**Fatty Acid Derivatives**											
Leukotriene D4 methyl ester	511.2812	4.14	M+H-H_2O, M+H	$C_{26}H_{42}N_2O_6S$	-4.81183	6.51	10.25	13.82	7.99	10.18	10.38
**Other Categories**											
4R-aminopentanoic acid	118.0868	0.50	M+H	$C_5H_{11}NO_2$	4.595853	-35.93	-96.57	-185.07	-399.83	-1370.72	-599.19

Cerebroside B	750.5449	7.89	M+H, M+Na	$C_{41}H_{77}NO_9$	-5.75781	15.77	13.21	15.72	9.02	8.59	7.46
N-docosahexaenoyl glutamine	457.3052	4.09	M+H-H_2O, M+H	$C_{27}H_{40}N_2O_4$	-1.85897	8.66	12.40	16.70	8.33	12.08	13.22
(1S,2S,3R,4R,5R,6S)-2,3,4,5,6-Pentahydroxycyclohexyl α-D-galactopyranosyl-(1->6)-α-D-galactopyranosyl-(1->6)-α-D-galactopyranoside	667.2331	3.72	M+H	$C_{24}H_{42}O_{21}$	5.900476	-268.24	-34.93	-26.99	-112.95	-97.28	-74.47
Phosphopantetheine	381.0822	0.48	M+Na, M+K	$C_{11}H_{23}N_2O_7PS$	-9.48272	-2.45	-3.55	-4.16	-3.05	-2.20	-2.68
Stachyose	689.2096	0.50	M+H-H_2O, M+H, M+Na	$C_{24}H_{42}O_{21}$	-2.22658	-2.89	-3.24	-3.01	-1.82	-1.56	-1.50

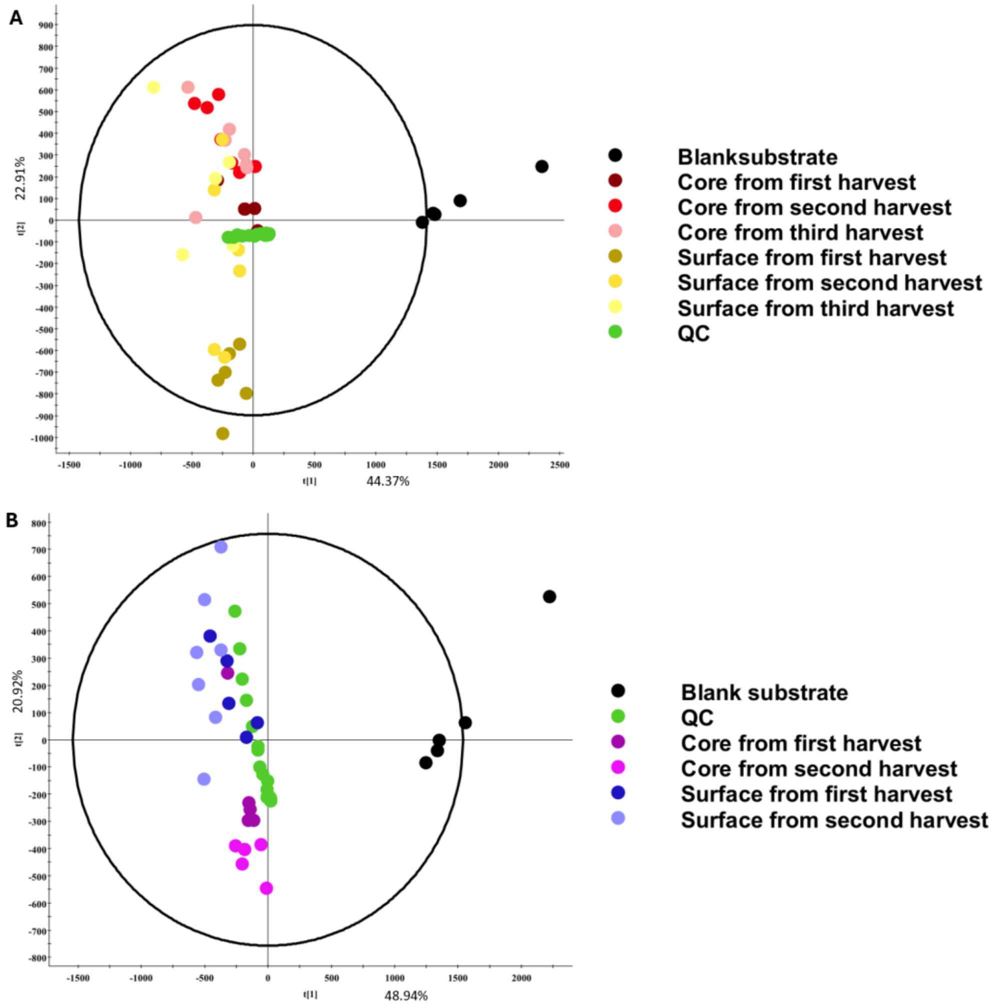

Figure S1. PCA analysis of blank substrate and spent pink oyster mushroom substrate (A) and spent pearl oyster mushroom substrate (B) (n = 5) grouping in different types and harvest of SMS.
